# Comparison of self-collected oral–nasal and mid-turbinate swabs to healthcare worker-collected nasopharyngeal swabs for the detection of SARS-CoV-2: a paired clinical evaluation

**DOI:** 10.1099/acmi.0.001148.v3

**Published:** 2026-07-15

**Authors:** Jodi Gilchrist, Kelly Waters, Sarah Marttala, Doris Williams, Kaitlyn Van Deventer, Keltie Baldwin, Alexandria Martindale, David Bulir, Mohammad Rubayet Hasan, Marek Smieja

**Affiliations:** 1 Research St. Joseph’s – Hamilton, Hamilton, ON, Canada; 2 Department of Health Research Methods, Evidence, and Impact, McMaster University, Hamilton, ON, Canada; 3 Department of Chemical Engineering, McMaster University, Hamilton, ON, Canada; 4 Department of Pathology and Molecular Medicine, McMaster University, Hamilton, ON, Canada; 5 Hamilton Regional Laboratory Medicine Program, St. Joseph’s Healthcare Hamilton, Hamilton, ON, Canada

**Keywords:** SARS-COV-2, PCR

## Abstract

Accurate detection of respiratory viruses is essential for infection control, patient management and public health response. Nasopharyngeal swabs (NPS), collected by healthcare workers (HWC-NPS), remain the gold standard for Severe Acute Respiratory Syndrome Coronavirus 2 (SARS-CoV-2) detection but require trained personnel and can be uncomfortable for patients. Self-collected swabs, such as oral–nasal swabs (SC-ONS) and mid-turbinate swabs, offer scalable alternatives suitable for mass testing. Previous mid-turbinate swab designs included a plastic stopper and were not compatible with automated laboratory testing. This study compared the performance of (i) SC-ONS, (ii) an automation-friendly, redesigned version of mid-turbinate swab (SC-MTS) and (iii) HWC-NPS in detecting SARS-CoV-2. Between April and June 2022, paired NPS, ONS and MTS samples were collected from 100 participants at a Coronavirus disease 2019 (COVID-19) assessment centre in Hamilton, Ontario. Samples were tested for SARS-CoV-2 by reverse-transcriptase PCR. Compared to HWC-NPS, SC-ONS demonstrated 82.1% sensitivity and 100% specificity, while SC-MTS showed 75.0% sensitivity and 100% specificity. Agreement with HWC-NPS was strong for both SC-ONS (κ=0.863) and SC-MTS (κ=0.804). Agreement between SC-ONS and SC-MTS was nearly perfect (κ=0.944). Cellular material yields as well as SARS-CoV-2 viral loads were lower for self-collected swabs than HWC-NPS, while viral load comparisons revealed no significant difference between SC-ONS and SC-MTS. Our study aligns with previous work demonstrating the use of self-collected mid-turbinate swabs for the detection of respiratory viruses, while also demonstrating the compatibility of the newly designed swabs with automated laboratory instruments. Our results, while conducted in a relatively small sample, suggest that self-collected ONS and MTS are reliable alternatives to HWC-NPS, offering practical, less invasive, automation-friendly options for large-scale respiratory virus surveillance and pandemic preparedness.

## Data Summary

In accordance with ethics review board approval, individual-level patient data are not available to maintain patient anonymity.

## Background

Since December 2019, there have been over 750 million confirmed Coronavirus disease 2019(COVID-19) cases and over 7 million COVID-19 deaths worldwide [1]. Accurate detection of respiratory viruses, including SARS-CoV-2, is crucial in limiting the spread of infection, implementing appropriate public health measures and managing patients who are severely or critically ill with respiratory viral infections. Currently, standard respiratory virus testing relies on the collection of nasopharyngeal swab (NPS) [2] specimens and amplification of viral genomic targets by real-time, reverse-transcriptase PCR.

While the sensitivity of NPS testing is high, collection must be performed by licensed healthcare professionals, and some patients may experience discomfort during the procedure. Non-NPS, self-collected methods of swabbing are ideal for scenarios where individuals do not require physician assessment. Over the course of the COVID-19 pandemic, self-swabbing has facilitated large-scale COVID-19 testing programmes at schools and workplaces and self-collected oral-nasal swabs (SC-ONS) are an adequate alternative to healthcare worker-collected NPS (HWC-NPS), with 90% sensitivity for detecting SARS-CoV-2 [3].

Additionally, mid-turbinate nasal (MT) swabbing has previously been shown to have 80–90% agreement with NPS swabbing for SARS-CoV-2 detection; however, most studies were performed using universal flocked swabs, and collection was performed by a healthcare provider [4,5]. Previously, Copan Italia developed a contoured mid-turbinate swab (MTS), designed specifically for self-swabbing with a stopper 5.5 cm along the shaft. The MTS consists of a tapered cone-shaped design, intended to sample a larger surface area of respiratory mucosa [6]. In previous studies, it was found to be comparable to HWC-NPS in terms of epithelial cell counts, and self-collection using this swab was able to detect several seasonal respiratory viruses with >70% sensitivity [6,7]. Due to the implementation of high-throughput robotic liquid handling systems to increase sample testing capacity during the SARS-CoV-2 pandemic, the swab was redesigned without the stopper to be compatible with laboratory automation. While the use of the MTS has been well-established in the literature, the redesigned MTS has not yet been evaluated on automated liquid handling instruments for the detection of SARS-CoV-2. In this study, we compare HWC-NPS, SC-ONS and self-collected MTS (SC-MTS) using the redesigned MTS for the detection of SARS-CoV-2.

## Methods

MTS, oral–nasal swab (ONS) and NPS samples were collected in parallel from patients presenting to a Hamilton COVID-19 assessment centre between April and June 2022, for a prospective comparison of the three sample types. Any individual presenting to the assessment centre for COVID-19 testing was eligible for the study. Ethical approval was received from the Hamilton Integrated Research Ethics Board, and verbal consent was obtained from all study participants. NPS samples were collected by a healthcare professional first in all cases and were retrieved from the regional laboratory following clinical testing for SARS-CoV-2. Participants were provided instructions for self-collection of ONS and MTS samples by a member of the research team. Supervised ONS samples were collected by first inserting the swab into the buccal area and rotating on each side of the mouth, followed by inserting the swab into the anterior nares and rotating. Self-collection of the MTS or ONS swab was alternated in a randomized fashion to account for possible sampling variability due to swab collection order [8]. All swabs were collected into McMaster Molecular Medium and tested for SARS-CoV-2 using a validated, laboratory-developed RT-PCR assay targeting the 5′-UTR and E- genes of SARS-CoV-2 and RNaseP, a human gene, used as a sample adequacy control. Quantitation was performed using an N1 standard (Exact Diagnostics) and an RNaseP standard (gBlock standard, Integrated DNA Technologies). Swabs were considered positive if two or more of the SARS-CoV-2 targets were positive with a CT value <40. The 5′ UTR and E-gene regions of SARS-CoV-2 with similar Ct cut-off values for positivity were well utilized for SARS-CoV-2 testing during the COVID-19 pandemic in Canadian laboratories [9]. Sensitivity and specificity of the ONS and MTS were calculated in comparison to the NPS as the gold standard collection method, and agreement was assessed using Cohen’s kappa coefficient. Formulas for the calculation of sensitivity, specificity and accuracy are available in Supplementary Material 1 (available in the online Supplementary Material). NPS samples that could not be retrieved for the research study were excluded from sensitivity, specificity and agreement calculations. Sensitivity and specificity of the MTS were also calculated in comparison to the ONS as the gold standard due to its widespread use. One-way ANOVA and paired t-tests were used to compare the mean number of cells collected (Log_10_RNaseP copies/reaction) and mean viral load (Log_10_N1 copies/reaction) for each swab type. Assumptions of normality were assessed using the Shapiro–Wilk test and visual inspection of Q-Q plots, while homogeneity of variance was evaluated using Levene’s test. All samples were processed on the Hamilton Microlab Star (Hamilton Company, Reno, NV) liquid handling instruments without the removal of the swabs before processing.

## Results

One hundred participants were enrolled in this clinical evaluation. Age and sex characteristics of the enrolled participants are available in Table 1. Overall, 28% (28/100) of participants tested positive by at least one swab type. Eighty-nine participants had all three swab types available for analysis; 11 NPS specimens were not available after standard-of-care testing. ONS and MTS swab pairs were available for all 100 patients (Table 1). For detection of SARS-CoV-2, compared to HWC-NPS, the sensitivity and specificity of SC-ONS were calculated to be 82.1% (95% CI=63.1, 93.9) and 100% (95% CI=94.1, 100), respectively, with an overall agreement of 94.4% (*κ*=0.86; 95% CI=87.5, 97.6, *P*<0.001) (Table 2). The sensitivity and specificity of the SC-MTS were calculated to be 75.0% (95% CI=55.1, 89.3) and 100% (95% CI=94.1, 100), respectively, compared to HWC-NPS, and overall agreement was 92.1% (*κ*=0.80, 95% CI=0.67, 0.94) (Table 3). Overall agreement between the SC-ONS and SC-MTS was 98.0% (95% CI=93.0, 99.5), *κ*=0.94 (95% CI=0.87, 1.00). Kappa values indicate strong agreement between SC-ONS and SC-MTS with HWC-NPS and near-perfect agreement between SC-ONS and SC-MTS for the detection of SARS-CoV-2.

**Table 1.** Characteristics of enrolled participants and overall swab testing results

Characteristic		No. (%)
Number of participants		100
Age (years)	Min-max	20–76
	Mean (sd)	39.4 (14.3)
	Median	36.5
Sex	Male (%)	23 (23)
	Female (%)	77 (77)
ONS SARS-CoV-2 result	Positive (%)	24 (24)
	Negative (%)	76 (76)
MTS SARS-CoV-2 result	Positive (%)	22 (22)
	Negative (%)	78 (78)
Number of participants with HCW-NPS available for testing		89
NPS SARS-CoV-2 result	Positive (%)	28 (31.5)
	Negative (%)	61 (68.5)

**Table 2.** Comparison of SARS-COV-2 results for different swab types

Statistic	NPS vs. ONS (*n*=89)		NPS vs. MTS (*n*=89)		ONS vs. MTS (*n*=100)	
	Value	95%** CI**	Value	95%** CI**	Value	95%** CI**
True positive	23	–	21	–	22	–
False positive	0	–	0	–	0	–
True negative	61	–	61	–	76	–
False negative	5	–	7	–	2	–
Sensitivity	82.1%	63.1% to 93.9%	75.0%	55.1% to 98.3%	91.67%	73.0% to 99.0%
Specificity	100.0%	94.1% to100.0%	100.0%	94.1% to100.0%	100.0%	95.26% to100.0%
Accuracy	94.4%	87.5% to 97.6%	92.1%	84.6% to 96.1%	98.0%	93.0% to 99.5%
PPV	100.0%	–	100.0%	–	100.0%	–
NPV	92.4	84.5% to 96.8%	89.7	84.5% to 96.8%	97.4%	90.98% to 99.31%
kappa	0.863	0.747 to 0.979, *P*<0.001	0.804	0.669 to 0.941, *P*<0.001	0.944	0.868 to 1.020, *P*<0.001

**Table 3.** Mean, minimum and maximum Ct values by gene targets for the three swab types

Swab	Measure (unit)	*N*	Mean (sd)	Minimum	Maximum
**SC-ONS (*n*=100)**	E-gene (CT)	24	24.78 (5.06)	16.07	35.50
	UTR (CT)	24	25.82 (5.00)	17.05	36.35
	N1 (CT)	24	23.17 (2.04)	15.55	32.95
	Log_10_N1 (copies/reaction)	24	4.72 (1.51)	1.83	6.99
	RNaseP (CT)	100	24.41 (2.04)	20.61	32.98
	Log_10_RNaseP (copies/reaction)	100	5.53 (0.78)	2.35	6.92
**SC-MTS (*n*=100)**	E-gene (CT)	22	23.45 (4.71)	16.74	32.74
	UTR (CT)	22	24.86 (4.64)	18.16	34.13
	N1 (CT)	22	22.24 (4.77)	15.57	33.45
	Log_10_N1 (copies/reaction)	22	5.00 (1.42)	1.69	6.89
	RNaseP (CT)	100	24.39 (1.59)	20.09	29.30
	Log_10_RNaseP (copies/reaction)	100	5.54 (0.58)	3.67	7.36
**HWC-NPS (*n*=89)**	E-gene (CT)	27	23.53 (8.04)	11.64	38.85
	UTR (CT)	26	24.14 (7.43)	13.16	39.16
	N1 (CT)	28	21.13 (8.31)	11.06	39.83
	Log_10_N1 (copies/reaction)	28	5.32 (2.45)	−0.19	8.31
	RNaseP (CT)	89	22.72 (1.82)	18.80	32.38
	Log_10_RNaseP (copies/reaction)	89	6.15 (0.66)	2.56	7.53

**Fig. 1.**
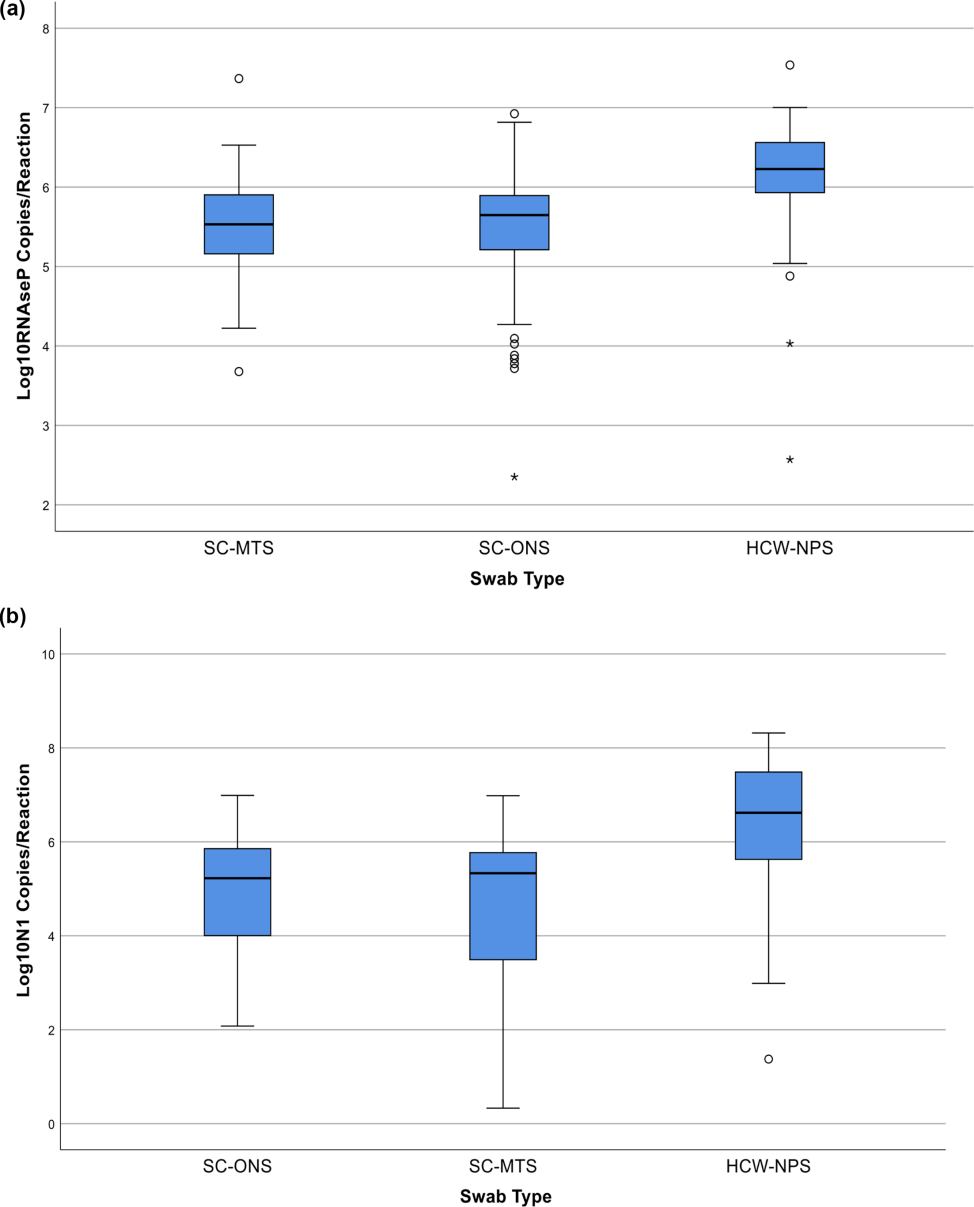
(a) RNaseP quantitation for different swab types in Log_10_RNaseP copies/reaction. Mean Log_10_RNaseP for SC-ONS, SC-MTS and HWC-NPS are 5.53±0.78, 5.54±0.58 and 6.16±0.66 copies/reaction, respectively. Mean difference in Log_10_RNaseP copies/reaction per Tukey’s Least Significant Difference (LSD) post hoc: SC-ONS vs. HWC-NPS=−0.63 (CI= −0.86,–0.39, *P*<0.001); SC-MTS vs. HWC-NPS=−0.62 (CI=−0.85,–0.39, *P*<0.001); SC-ONS vs. SC-MTS=−0.005 (CI=−0.23, 0.22, *P*=0.998). Circles (○) represent mild outliers calculated as greater than 1.5 times the interquartile range above Q3 or below Q1. Asterisks (*) represent extreme outliers calculated as greater than three times the interquartile range above Q3 or below Q1. (b) SARS-CoV-2 viral load (based on Ct values for N1) by swab types in Log_10_N1 copies/reaction. Mean Log_10_N1 for SC-ONS, SC-MTS and HWC-NPS are 4.72±1.51, 5.00±1.42 and 5.32±2.45 copies/reaction, respectively. Mean difference in Log_10_N1 copies/reaction by paired t-test: SC-ONS vs. HWC-NPS=−1.49 (95% CI=−2.11, –0.88, *P*<0.001); SC-MTS vs. HWC-NPS=−1.48 (95% CI=−2.11, –0.78, *P*<0.001); SC-ONS vs. SC-MTS=−0.02 (95% CI=−0.28, 0.4, *P*=0.870). Circles (○) represent mild outliers calculated as greater than 1.5 times the interquartile range above Q3 or below Q1.

To assess whether the three swab types collected equivalent mean amounts of cellular material, a one-way ANOVA was performed using Log_10_RNaseP copies/reaction as the dependent variable and swab type as the independent variable. Analysis revealed that there was a statistically significant difference in mean Log_10_RNaseP values between at least two groups [*F*(2, 286)=27.0, *P*<0.001]. Post hoc Tukey’s Honestly Signficant Difference (HSD) analysis found that the mean Log_10_RNaseP value was significantly different between both SC-ONS and HWC-NPS (−0.63, 95% CI=−0.86, –0.39, *P*<0.001) and SC-MTS and HWC-NPS (−0.62, 95% CI=–0.85, –0.39, *P*<0.001). No statistically significant difference was observed between the mean Log_10_RNaseP value of the SC-ONS and SC-MTS (−0.01, 95% CI=−0.23, 0.22, *P*=0.998) (Table 3, Fig. 1a). Subsequent paired t-tests revealed similar findings with statistically significant differences in mean Log_10_RNaseP copies/reaction between both SC-ONS and SC-MTS compared to HWC-NPS, but not between SC-ONS and SC-MTS.

One-way ANOVA comparing mean viral load of the three swab types revealed no statistically significant difference between mean Log_10_N1 values in any of the pairings [*F*(2,72)=0.725, *P*=0.488]. However, a subsequent paired t-test analysis revealed a statistically significant difference in mean Log_10_N1 copies/reaction between SC-ONS and HWC-NPS (−1.49, 95% CI=−2.11, –0.88, *P*<0.001) as well as SC-MTS and HWC-NPS (−1.48, 95% CI=−2.11, –0.78, *P*<0.001). No statistically significant difference was observed for mean Log_10_N1 values between SC-ONS and SC-MTS (−0.02, 95% CI=−0.28, 0.24, *P*=0.870) (Table 3, Fig. 1b).

## Discussion

While NPS sampling remains the gold standard for detection of SARS-CoV-2 and other respiratory viruses, collection is limited to healthcare workers and can be uncomfortable for the patient. SC-ONSs have previously been shown to be an adequate alternative for at-home or resource-limited swabbing and have been implemented into practice. Mid-turbinate swabbing has also been shown to be a good alternative to nasopharyngeal swabs for the detection of respiratory viruses, but previous studies have included swabs collected by a healthcare worker [4,5]. Here, we support findings from previous studies, which show SC-MTS and SC-ONS to be acceptable alternatives to HWC-NPS and demonstrate the utility of the automation-friendly MTS [10,11].

In this study, we demonstrate that SC-ONS and SC-MTS have good agreement with HWC-NPS for the detection of SARS-CoV-2. Further, SC-ONS and SC-MTS had near-perfect agreement for the detection of SARS-CoV-2 when compared to one another. Comparison of means analysis showed no statistically significant difference in the amount of cellular material collected by SC-ONS and SC-MTS. However, HWC-NPS collected a significantly higher (*P*<0.001) amount of cellular material when compared to both the SC-ONS and SC-MTS, which may be due to the deeper nature of the nasopharyngeal swab, although this did not contribute to any differences in positivity for the RNaseP target. When comparing viral loads, statistically significant differences were seen when comparing HWC-NPS and SC-ONS and HWC-NPS and SC-MTS, but only in paired specimens. Lower viral loads observed in SC-ONS and SC-MTS may contribute to the slightly lower sensitivity observed in these swab types.

While SARS-CoV-2 has now shifted from a pandemic to an endemic virus, ongoing surveillance remains important, especially when coupled with testing for additional pathogens during the respiratory season. Given the shared nature of specimen collection and testing for many respiratory bacteria and viruses, the findings of this study could be expanded to the detection of other targets, including emerging pathogens. Additionally, the COVID-19 pandemic has taught us the importance of preparedness and redundancy. At the height of the pandemic, swabs were in short supply and labs across the world were compelled to use whatever swabs were available. Many of these swabs were not compatible with high-capacity automated instruments, which meant that an upfront manual step, such as sample aliquoting or swab removal, was required. The newly designed mid-turbinate swab (SC-MTS) is compatible with the Hamilton Microlab STAR liquid handling instruments, allowing for high-throughput testing. This is an important feature as automation can reduce turnaround times and increase the scalability of testing. Further, as previous studies evaluating mid-turbinate swabs consisted of swabs collected by a healthcare professional [4,5], we demonstrate the potential use of SC-MTS for a future pandemic scenario.

One limitation observed in this study was the relatively low number of SARS-CoV-2-positive specimens and wide confidence intervals in sensitivity calculations for the SC-ONS and SC-MTS. Further, the study was conducted in a single centre, and generalizability may be affected due to the small sample size and demographic skew towards female participants. Additionally, for several participants, the HWC-NPS was not available for further testing after standard-of-care. This reduced the sample size for pairwise comparisons.

Overall, self-collection using the redesigned MTS may be an adequate alternative to an HWC-NPS since it can be self-collected with less discomfort. Self-collection using the redesigned MTS is shown to be a comparable alternative to the SC-ONS, which is currently in widespread use. While testing requirements for COVID-19 have decreased, the insights from this study may still apply to novel variants or other respiratory viruses in a future pandemic scenario.

## Supplementary material

10.1099/acmi.0.001148.v3 Supplementary Material 1.

## Abbreviations

HWC-NPS: healthcare worker-collected NPS
MT: mid-turbinate nasal
MTS: mid-turbinate nasal swab
NPS: nasopharyngeal swab
ONS: oral–nasal swab
SC-MTS: self-collected mid-turbinate swab
SC-ONS: self-collected oral–nasal swab
