# Supplementary material for: Comparison of self-collected oral–nasal and mid-turbinate swabs to healthcare worker-collected nasopharyngeal swabs for the detection of SARS-CoV-2: a paired clinical evaluation
Source: Access Microbiol. 2026 Jul 15;8(7):001148.v3. doi: 10.1099/acmi.0.001148.v3 (PMC13380853; doi:10.1099/acmi.0.001148.v3)
Supplement: Supplementary Material 1. [file acmi-8-01148-s001.pdf]

Comparison of Self-Collected Oral-Nasal and Mid-turbinate Swabs to Healthcare Worker-Collected Nasopharyngeal Swabs for the Detection of SARS-CoV-2: A Paired Clinical Evaluation – Supplementary File S1

Formulas for sensitivity, specificity and accuracy calculations:

Sensitivity = $\frac{True\ Positives}{(True\ Positives + False\ Negatives)}$

Specificity = $\frac{True\ Negatives}{(True\ Negatives + False\ Positives)}$

Accuracy = $\frac{True\ Positives + True\ Negatives}{(True\ Positives + True\ Negatives + False\ Positives + False\ Negatives)}$
